# Menthacarin for long-term treatment of functional dyspepsia – Results from a clinical trial follow-up

**DOI:** 10.1055/a-1823-1333

**Published:** 2022-06-13

**Authors:** Martin Storr, Berenike Stracke

**Affiliations:** 1 Zentrum für Endoskopie, Starnberg, Germany und Medizinische Klinik und Poliklinik II, Klinikum Großhadern, München, Germany; 2 Global Medical Affairs, Dr. Willmar Schwabe GmbH & Co. KG, Karlsruhe, Germany

**Keywords:** Funktionelle Dyspepsie, Follow-up-Studie, Kümmelöl, Pfefferminzöl, Menthacarin, Functional Dyspepsia, Follow-up Study, Caraway oil, Peppermint oil, Menthacarin

## Abstract

**Background** Menthacarin was shown to be effective and safe in clinical trials in patients with functional dyspepsia (FD). Long-term treatment results have not been reported yet.

**Methods** An open-label, 11-month follow-up (FU) was offered to FD patients who had undergone treatment with Menthacarin (1 gastro-resistant capsule b.i.d. vs. placebo (PL)) in a 4-week, double-blind, clinical trial. During FU, all patients (former verum and PL) were treated with 1 gastro-resistant capsule Menthacarin b.i.d. Main outcomes were the changes in pain intensity and severity of sensation of pressure, heaviness, and fullness from original baseline and global improvement.

**Results** 70 patients were included in the analyses (former Menthacarin group: 36, former PL group: 34). At the end of the PL-controlled study phase, all 3 main efficacy variables were statistically significantly improved in the Menthacarin group compared to PL. In the FU phase, former PL patients started to improve under Menthacarin treatment towards the outcomes seen in the former Menthacarin group (alignment at approximately 6 months), while former Menthacarin patients showed sustained or even continuously improved outcomes by month 12. At study end, more than 90% of patients were “much or very much improved" in both groups. Menthacarin treatment was well tolerated.

**Conclusions** The favorable effects seen in the FU period suggest that Menthacarin is a valuable treatment option in FD patients who require symptomatic treatment also in the longer term for up to 12 months.

## Zusammenfassung

**Hintergrund** Menthacarin hat sich in klinischen Studien bei Patienten mit funktioneller Dyspepsie (FD) als wirksam und sicher erwiesen. Langzeitergebnisse einer Behandlung wurden bisher nicht berichtet.

**Methodik** FD-Patienten, die in einer 4-wöchigen klinischen Doppelblindstudie mit Menthacarin (1 gastroresistente Kapsel b.i.d. vs. Placebo [PL]) behandelt worden waren, wurde ein offenes, 11-monatiges Follow-up (FU) angeboten. Während des FU erhielten alle Patienten (sowohl ehemalige Verum- als auch PL-Patienten) 1 magensaftresistente Kapsel Menthacarin b.i.d. Wichtigste Zielparameter waren die Veränderung der Schmerzintensität und des Schweregrads des Druck-, Schwere- und Völlegefühls sowie die globale Verbesserung.

**Ergebnisse** Die Analyse umfasste insgesamt 70 Patienten (36 ehemals Menthacarin, 34 ehemals PL). Alle 3 Hauptwirksamkeitsvariablen waren in der Menthacarin-Gruppe am Ende der PL-kontrollierten Studienphase im Vergleich zu PL statistisch signifikant verbessert. In der FU-Phase begannen sich die vormaligen PL-Patienten unter Menthacarin-Behandlung zu verbessern und sich den Ergebnissen der vormaligen Menthacarin-Gruppe anzunähern (Angleichung nach etwa 6 Monaten), während die letztere Gruppe bis Monat 12 eine anhaltende oder fortschreitende Besserung zeigte. Am Studienende waren über 90% der Patienten beider Gruppen stark oder sehr stark verbessert. Die Menthacarin-Behandlung war gut verträglich.

**Schlussfolgerungen** Die in der FU-Periode beobachteten günstigen Effekte legen nahe, dass Menthacarin eine wertvolle Therapieoption bei FD-Patienten ist, welche eine symptomatische Behandlung benötigen, auch bei einer längerfristigen Einnahme über bis zu 12 Monate.

## Introduction

With 20% of the global population suffering from symptoms of dyspepsia, there is a significant economic impact of dyspeptic symptoms to health services and society 1 . In the West, and increasingly in other parts of the world, functional dyspepsia (FD; also referred to as “non-ulcer dyspepsia”) is the most common cause of dyspeptic symptoms, which include, e.g., pain, cramping, bloating and fullness.

Generally, the term “dyspepsia” refers to pain or discomfort centered in the upper abdomen, with discomfort referring to a subjective negative (or aversive) feeling that is distinct from pain. Discomfort may include several specific bothersome but non-painful symptoms, such as early satiety, fullness, bloating, and nausea 2 .

The definition of FD has evolved over time. In order to standardize the diagnosis and treatment of functional gastrointestinal disorders (FGIDs; now also called “disorders of gut-brain interaction” (DGBI) 3 ) and to identify more homogeneous patients for inclusion in clinical trials, the Rome criteria were established in 1990 and have since been modified thrice 3 . Whereas, e.g., the Rome II diagnostic criteria defined FD as recurrent or persistent dyspepsia (pain or discomfort centered in the upper abdomen) for at least 12 weeks, non-consecutive, within the preceding 12 months and sorted the subtypes on the basis of the predominant symptoms (ulcer-like dyspepsia, dysmotility-like dyspepsia, and unspecified dyspepsia) 4 , the current Rome IV criteria define FD as any combination of the 4 symptoms postprandial fullness, early satiety, epigastric pain, and epigastric burning that are severe enough to interfere with the usual activities and occur at least 3 days per week over the last 3 months with an onset of at least 6 months earlier 5 . It subclassifies FD into two main categories and a combination of both: a) “postprandial distress syndrome” (PDS), characterized by meal-induced dyspeptic symptoms suggestive of a motility disturbance; b) “epigastric pain syndrome” (EPS), referring to epigastric pain or epigastric burning which does not necessarily occur after meal ingestion, may occur during fasting and can be even improved by meal ingestion, is reminiscent of the clinical features typical of a peptic ulcer, and needs to be distinguished from gastro-esophageal reflux disease; c) overlapping PDS and EPS, characterized by meal-induced dyspeptic symptoms and epigastric pain or burning 6 .

The diagnosis of FD is a pure diagnosis of exclusion, involving careful history taking, review of alarm symptoms, physical examination and final consideration of the diagnosis 7 .

In a recent analysis performed in the USA, Canada, and the UK, the prevalence of FD diagnosed according to Rome IV was 12% in the USA and 8% in Canada and the UK, respectively 8 . The subtype distribution was 61% for PDS, 18% for EPS, and 21% for the overlapping variant with both syndromes; this pattern was similar across the countries. Importantly, subjects with FD had significantly greater health impairment and health-care usage than those without FD 8 .

The pathophysiology of FD is multifactorial and still not fully understood 7 . Gastroduodenal motor and sensory dysfunction, as well as impaired mucosal integrity, low-grade immune activation, and dysregulation of the gut-brain axis have all been implicated. Increased permeability of the mucosal barrier exposes the submucosal immune system to luminal noxious agents. Beyond *H. pylori* -associated gastritis, duodenal eosinophilia has been reported in FD patients, suggesting potential new therapeutic approaches. Infections, stress, duodenal acid exposure, smoking, and food allergy have all been implicated in the pathogenesis of duodenal mucosal inflammatory and permeability changes. Post-infectious dyspepsia has been reported, although it seems to be short-lived, compared to post-infectious irritable bowel syndrome (IBS). Psychosocial disorders may also play a role: anxiety, depression, neuroticism, as well as physical and emotional abuse and difficulty in coping with life events, are frequent among dyspeptic patients. A bidirectional relationship probably exists between gut and psyche: studies on the natural history of FGIDs suggest that patients affected by FD and IBS are particularly prone to develop psychological problems 6 .

Due to the multifactorial etiology of FD, treatment options are widespread, all aimed at symptom control. Disease management starts with comprehensive patient’s history taking. As there seems to be little evidence for an improvement of symptoms following patient education about dietary and lifestyle considerations, medical therapy remains the main component of treatment with psychotherapy as well as drug treatment with acid-reducing proton pump inhibitors (PPIs), *H. pylori* eradication treatment, tricyclic antidepressants, and phytotherapeutics being the main medical treatment approaches 7 . However, dysmotility symptoms in the sense of a PDS seem not to respond to PPI, and the use of prokinetics in daily routine is limited due to the market withdrawal of cisapride (due to cardiotoxicity) and the rare, but potentially severe adverse effects of domperidone and metoclopramide, especially with long term use 9 .

Phytotherapeutics have been used in medicine for decades, and numerous placebo-controlled trials have shown a significantly positive effect compared to placebo in the treatment of FD 10 11 . Combination preparations are often used to treat FD. Mostly, these are fixed combinations of peppermint and caraway oil or mixtures of bitter candytuft, wormwood, gentian, and angelica root, usually in combination with spasmolytic and sedative extracts such as chamomile, peppermint, caraway, and lemon balm. Phytotherapeutics exert a spasmolytic tonus-stimulating and/or sedative effect on the gastrointestinal tract, and this may relieve the symptoms of FD 10 . In the wake of numerous positive placebo-controlled studies, phytotherapy is now recommended in German and international guidelines for use in FGIDs, in particular for FD and IBS 5 9 12 .

A number of randomized, controlled trials (RCTs) 13 14 15 16 17 have shown the efficacy and safety of the peppermint oil/caraway oil combination Menthacarin (Menthacarin is the active agent of the product *Carmenthin bei Verdauungsstörungen* (Dr. Willmar Schwabe GmbH & Co. KG, Karlsruhe, Germany)) in the treatment of FD. Menthacarin is a proprietary combination of essential oils of a specified quality from Mentha x piperita L. and Carum carvi. The pharmacological effect of these two essential oils on the digestive process and gastrointestinal motility have already been the subject of careful non-clinical and clinical studies. In various basic science studies as well as in human pharmacological studies, a spasmolytic action on smooth muscle was demonstrated for peppermint oil due to its calcium-antagonistic properties, while caraway oil shows antimeteoric and choleretic effects and decreases the muscular tone 18 19 20 21 22 23 . Moreover, peppermint oil has shown choleretic 24 and direct analgesic properties through inhibition of voltage-gated sodium channels 25 26 and activation of the cold receptor TRPM8 27 .

In two placebo-controlled, double-blind clinical trials investigating FD treatment with Menthacarin over 4 weeks 13 15 , a statistically significant advantage was shown with Menthacarin treatment compared to placebo. In addition, a post hoc analysis showed that Menthacarin had beneficial effects in both *H. pylori* -positive and *H. pylori* -negative patients 28 . Moreover, treatment with Menthacarin in patients with FD was shown to be comparable to treatment with the prokinetic cisapride in a 4-weeks trial 14 . Equivalence of the gastro-resistant Menthacarin formulation vs. an immediate-release formulation over 4 weeks in patients with FD or IBS has been shown 16 . In a third placebo-controlled study, 114 patients with FD were treated with Menthacarin over 4 weeks and separated into those with predominant EPS or PDS. In this study, the validated Nepean Dyspepsia Index (NDI), which was used as primary outcome measure, significantly improved with Menthacarin treatment compared to placebo 17 . The results of a subsequent 8-week open follow-up trial part also showed statistically significant differences in favor of Menthacarin in the NDI subscores for pain and discomfort, respectively, and in the quality of life of the patients 29 .

Long-term treatment experience with Menthacarin administered over a 12-month period in FD has not been reported so far. Here, we present results from patients entering an open-label, follow-up period of the double-blind, placebo-controlled clinical trial performed and reported by May and colleagues 15 . In this follow-up period, all participating patients (former verum and placebo) received Menthacarin treatment for 11 months. The favorable main results for the whole study population of the preceding double-blind, placebo-controlled trial phase have been published elsewhere 15 . The results for those patients entering the follow-up study, covering the double-blind treatment phase plus the 11 months treatment with Menthacarin (resulting in an up to 12 months treatment with Menthacarin), are presented here.

## Methods

### Overall study design

The study was an open-label follow-up of a randomized, double-blind, placebo-controlled study performed in 7 private practice sites in Germany.

The placebo-controlled phase of the study was designed to demonstrate superiority of Menthacarin vs. placebo in a confirmatory fashion primarily in terms of pain reduction over a treatment period of 29 days. Following written informed consent of the patient, screening examinations for the double-blind period took place during a period of up to 7 days before baseline assessment. Moreover, an adequate wash-out for drugs not allowed according to the study protocol (please refer to 15 ) was performed. During this period, no trial-specific medication was administered. Patients complying with the inclusion and exclusion criteria were then randomized to Menthacarin or placebo for a treatment period of 4 weeks. An intermediate visit took place on day 15 followed by the final evaluation of the double-blind phase at day 29.

Patients who had completed the double-blind, placebo-controlled, randomized clinical trial were then offered the opportunity to voluntarily undergo follow-up treatment with Menthacarin for a further 11-months. Patients who agreed to participate had to give written informed consent for entering this open-label study part. Assessment during follow-up treatment took place every other month from month 4 (post baseline) onwards (i.e., months 4, 6, 8, 10, and 12).

### Ethics

The study was conducted in accordance with the principles of the Declaration of Helsinki and Good Clinical Practice (GCP), and all local regulatory requirements. The study protocol was approved by an independent Ethics Committee and written informed consent (IC) was obtained from all patients prior to initiation of any study-related procedures.

### Eligibility criteria

Male or female adult patients with a secured diagnosis of FD and a current episode of FD lasting for at least 14 days could be enrolled. FD was defined as diffuse, unspecific, variable, moderately intense epigastric pain with at least 4 score points on a visual analogue scale (VAS, 0–10 cm), at least one additional dyspeptic key symptom, and organ pathology excluded by in-depth clinical examination including abdominal sonography, esophagogastroduodenoscopy, rapid urease test, and stool test for occult blood. Major exclusion criteria comprised the presence of concomitant disorders such as congestive heart failure (NYHA III-IV), severe renal or hepatic insufficiency, malignancies, severe metabolic and other disorders that could cause gastrointestinal symptoms, severe functional disorders other than FD, severe diarrhea, history of major gastrointestinal surgery, verified organic etiology of gastrointestinal symptoms (e.g., obstruction of bile ducts, cholelithiasis, cholecystitis, reflux esophagitis, duodenal or ventricular ulcer), or any suspicion of an organic genesis. In addition, concomitant medications that were expected to interact with the assessment of the study evaluation criteria (e.g., prokinetics, agonists and antagonists of gastric hormones, acid-reducing drugs, bismuth preparations, benzodiazepines, laxatives, opioids, non-steroidal antiphlogistics, other herbal preparations acting on the gastrointestinal tract) were to be discontinued at least 1 week prior to study enrollment.

### Study treatments

Patients eligible for study inclusion were then randomized in a 1:1 ratio (block size of 4) to a 4-week treatment (day 1 to day 29) with either 2 gastro-resistant capsules Menthacarin per day (one in the morning and one at lunch time, 30 min before the meal) or matching placebo (for more information on randomization and blinding please refer to 15 ). Menthacarin is a proprietary combination of essential oils of a specified quality from Mentha x piperita L. (90 mg peppermint oil WS 1340) and Carum carvi (50 mg caraway oil WS 1520). Patients entering the follow-up study continued the same active treatment (in case of patients of the former active treatment group) or were switched from placebo to active treatment (in case of patients of the former placebo group). The treatment was then continued open label up to month 12, i.e., for a further 11 months after the double-blind period. Medication compliance was checked by means of pill count.

A more detailed description of study methods for the double-blind treatment phase of the trial can be found elsewhere 15 .

### Criteria for evaluation

The dyspepsia-type of the patient (dysmotility-type dyspepsia, ulcerous dyspepsia, idiopathic/essential dyspepsia) was diagnosed by the investigator at baseline. Treatment effects were assessed at each visit (day 15 and 29, months 4, 6, 8, 10, and 12) by obtaining the patient’s ratings of intensity of epigastric pain and sensation of pressure, heaviness and fullness (PHF) with a standardized self-rating questionnaire using a continuous 10 cm VAS (0=“no pain/absent” to 10=“maximum intensity”) and by the investigator’s rating of global clinical improvement (Clinical Global Impressions, CGI item II), which yields values between 1 (“very much improved”) and 7 (“very much worse”). For additional outcome variables, the patients recorded the pain frequency on a 10 cm VAS of the type described above (0=“never” to 10=“permanent”), and the investigators assessed the patients’ condition on CGI item I (severity of illness; 1=“not at all ill” to 7=“among the most extremely ill patients”) and the therapeutic effect (item III). In addition, a “Dyspeptic Discomfort Score” (DDS) according to Madisch and colleagues 14 was calculated, which summarizes the ordinal-scaled and weighted results of various parameters from the areas of dyspeptic symptoms (FD score), intestinal symptoms (irritable bowel symptom score), and vegetative symptoms (autonomic symptom score). Values of the final DDS score range from 0 (minimum impairment) to 84 (maximum impairment). The safety of investigational treatment was assessed by documentation of any adverse events (AEs) that occurred during the study.

Primary outcomes of the double-blind period were the absolute intra-individual changes in the patient ratings of (i) pain intensity and (ii) sensation of PHF between day 0 (baseline value of each group) and day 29, and (iii) the global improvement (CGI item II) at day 29. Secondary outcomes were severity of illness (CGI item I), therapeutic effect (CGI item III), pain frequency, and DDS. These variables were continuously assessed beyond day 29 through the end of the follow-up period at month 12.

### Statistical analyses

All statistical analyses of the treatment effect data collected during the follow-up period were purely descriptive and performed on the intent-to-treat (ITT) population based on patients who had at least one documentation of the outcome variables in the follow-up period (follow-up ITT). Protocol deviations were no longer documented. Missing values for patients who withdrew during the follow-up period were imputed using the “last observation carried forward” (LOCF) approach. For the exploratory testing of potential group differences in the main outcome variables, the 2-sided Wilcoxon-Mann-Whitney-test was performed based on an alpha-level of 5%. The ordinally-scaled CGI items I and II were analyzed both as continuous and categorical data.

## Results

### Disposition of patients

**Fig. 1:**
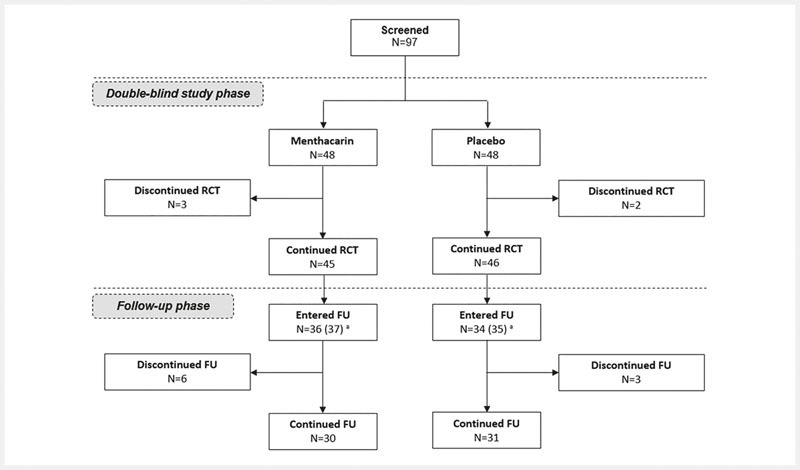
Patient disposition from original study start through month 12. FU = Follow-up; RCT= Randomized controlled trial; a: Two patients agreed to continue the study in the follow-up phase but did not contribute any data. These patients were included in the follow-up SAF (N=72) but not in the follow-up ITT (N=70).

A total of 97 patients were screened for the double-blind treatment phase. Of these, one patient discontinued trial participation prior to the first administration of the study drug due to violation of an exclusion criterion. Therefore, 96 patients (48 in each treatment group) were randomized to double-blind treatment ( Fig. 1 ). Five patients (3 in the Menthacarin group and 2 in the placebo group) discontinued the double-blind phase prematurely, while the remaining 45 and 46 patients, respectively, continued the study through day 29. Out of these 91 patients, 72 patients consented to participate in the follow-up period, and 70 patients finally had at least one observation in the follow-up period documented. These 70 patients (36 in the former Menthacarin group and 34 in the former placebo group) constituted the ITT population for the present study analyses. A total of 9 patients dropped out during follow-up; the drop-out reasons among the 6 patients in the former Menthacarin group were worsening of symptoms and dissatisfaction with study drug (2 patients), lack of compliance and revocation of informed consent (2 patients), revocation of informed consent due to symptom-free condition (1 patient), and premature termination of treatment due to a planned hospitalization (1 patient); drop-out reasons among the 3 patients in the former placebo group were premature termination/revocation of informed consent due to symptom-free condition (2 patients) and revocation of informed consent due to other clinical condition (diabetes mellitus) (1 patient). Finally, 30 and 31 patients in the former Menthacarin and placebo group, respectively, had completed the planned 11 months of follow-up.

### Treatment compliance

The mean treatment duration in the follow-up period from day 29 onwards was 297.7 ± 63.4 days overall, 293.2 ± 65.0 days in the former Menthacarin group (range: 115 to 410 days) and 302.7 ± 62.3 days in the former placebo group (range: 54 to 392 days).

The investigators reported regular intake of the study medication in the majority of patients. The mean medication compliance was 100.0% in the former Menthacarin group and 98.4% in the former placebo group. Apart from 1 patient in the former Menthacarin group (medication compliance of 120.2%) and 1 patient in the former placebo group (medication compliance of 78.8%), all remaining patients showed a medication compliance that ranged between 80 and 120%.

### Demographic and other baseline characteristics

Baseline characteristics of the patients of the follow-up period are summarized in Table 1 . The patients’ mean age was 52.1 ± 18.1 years in the former Menthacarin group and 50.6 ± 11.1 years in the former placebo group; most patients of either treatment group were women (77.8% and 64.7%, respectively). Patients were mainly diagnosed with idiopathic/essential dyspepsia (44.4% and 52.9%, respectively), followed by dysmotility-type dyspepsia (38.9% and 35.3%, respectively) and ulcerous dyspepsia (16.7% and 11.8%, respectively). The descriptive data did not point to relevant baseline imbalances between the 2 treatment groups.

**Table:** **Table 1** Baseline (Day 0) demographic and clinical characteristics of trial patients also taking part in the follow-up period (follow-up ITT).

Variable		Former Menthacarin group (N=36)	Former placebo group (N=34)
**Sex, n (%)**			
Female		28 (77.8)	22 (64.7)
Male		8 (22.2)	12 (35.3)
**Age (years)**	Mean ± SD	52.1 ± 18.1	50.6 ± 11.1
**Body height (cm)**	Mean ± SD	168.5 ± 9.1	169.9 ± 9.5
**Body weight (kg)**	Mean ± SD	70.5 ± 13.8	75.3 ± 15.0
** Intensity of pain ^a^**	Mean ± SD	6.7 ± 1.3	6.9 ± 1.0
** Intensity of PHF symptoms ^a^**	Mean ± SD	6.7 ± 1.3	6.8 ± 1.1
**Diagnoses, n (%)**			
Dysmotility-type dyspepsia ^b^		14 (38.9)	12 (35.3)
Ulcerous dyspepsia ^c^		6 (16.7)	4 (11.8)
Idiopathic/essential dyspepsia ^d^		16 (44.4)	18 (52.9)
PHF=Pressure, heaviness and fullness; SD=Standard deviation. Note: Exploratory 2-sided tests (Chi-square test or Wilcoxon-Mann-Whitney-test) ranged between p=0.16 (body weight) and p=0.88 (intensity of PHF symptoms) and thus were not indicative of nominally significant group differences. a: Measured on a 10 cm visual analog scale (VAS). b: Mainly dyspeptic symptoms, e.g., sensation of fullness. c: Mainly epigastric symptoms, e.g., pain. d: All other types or combinations of symptoms.			

### Outcome measures

At the end of the double-blind trial phase, intensity of pain, sensation of PHF, and CGI item II were statistically significantly improved with Menthacarin treatment compared with placebo. Likewise, nominally significant improvements compared with placebo were seen for DDS and continuous severity of illness, and descriptively better outcomes were achieved in CGI item III.

**Fig. 2:**
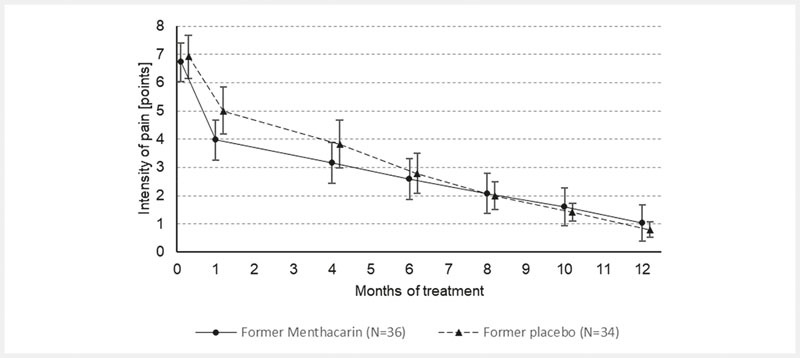
Pain intensity – Time course from baseline through month 12 (follow-up ITT; means and 95% CI). Note: Pain intensity was measured on a 10 cm VAS anchored from 0 = “no pain” to 10 = “extremely severe pain”. Provided are the arithmetic means based on 36 patients in the former Menthacarin group and 34 patients in the former placebo group (treated with placebo until day 29, then switched to Menthacarin). Vertical error bars indicate the 95 %-confidence intervals for the respective mean.

**Fig. 3:**
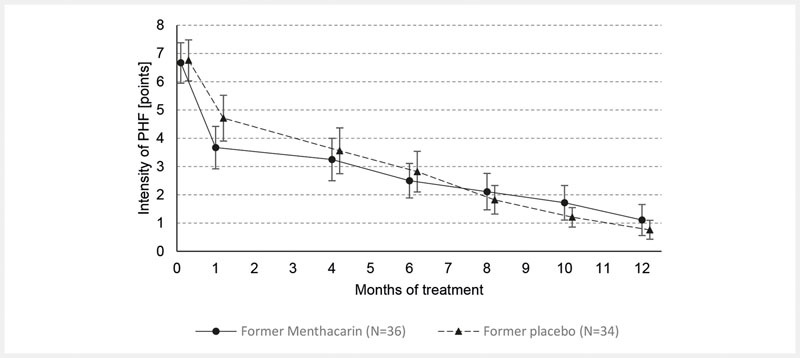
Sensation of pressure, heaviness or fullness – Time course from baseline through month 12 (follow-up ITT; means and 95% CI). Note: Intensity of sensation of pressure, heaviness or fullness was measured on a 10 cm VAS anchored from 0 = “absent” to 10 = “severe”. Provided are the arithmetic means based on 36 patients in the former Menthacarin group and 34 patients in the former placebo group (treated with placebo until day 29, then switched to Menthacarin). Vertical error bars indicate the 95 %-confidence intervals for the respective mean.

**Fig. 4:**
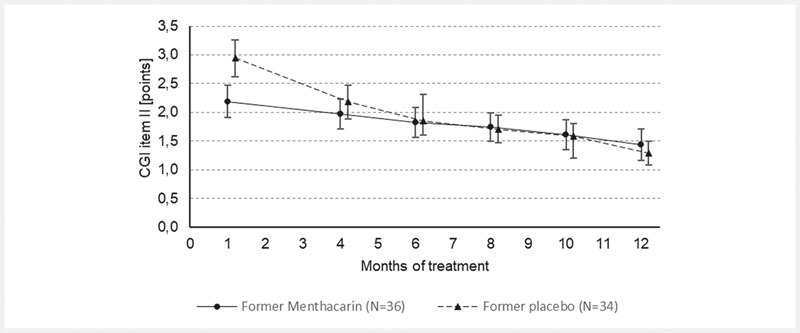
CGI item II – Development from the end of the placebo-controlled trial (month 1) throughout the follow-up trial part (months 4, 6, 8, 10, and 12) (follow-up ITT; means and 95% CI). Note: Provided are the arithmetic mean values in continuous GCI item II over time based on 36 patients in the former Menthacarin group and 34 patients in the former placebo group (treated with placebo until day 29, then switched to Menthacarin). Values <4 indicate improvement (the lower the value, the stronger the improvement). Vertical error bars indicate the 95 %-confidence intervals for the respective mean value.

During the follow-up period through month 12, the descriptive analyses of intensity of pain, sensation of PHF, and CGI item II showed a continuously ongoing improvement in the former Menthacarin group and a steadily converging improvement in the former placebo group. In the latter group, similar treatment results as in the former Menthacarin group were achieved by month 6 at the latest. The improvements in either group were ongoing until the last study visit at month 12 ( Fig. 2 , Fig. 3 , and Fig. 4 ).

In the former Menthacarin group, patients had a mean pain intensity baseline value of 6.72 ± 1.32 points and continued to improve from 3.97 ± 1.98 points at day 29 to 1.03 ± 1.86 at month 12 ( Fig. 2 ), resulting in a difference of 5.69 ± 2.16 points from baseline at month 12 ( Table 2 ). Likewise, the mean pain intensity in the former placebo group improved from 6.91 ± 1.03 points at baseline to 5.00 ± 2.22 points at day 29 to 0.79 ± 0.77 at month 12 (difference: 6.12 ± 1.25 points) and converged with the former Menthacarin group approximately at month 6 ( Fig. 2 ; Table 2 ). At the first follow-up evaluation time point (month 4), statistical differences between treatment groups could no longer be observed.

**Table:** **Table 2** Pain Intensity – Difference from baseline at different time points (follow-up ITT; means and 95% CI).

Time Point	Former Menthacarin group (N=36)	Former placebo group (N=34)
**Day 29**	2.75 [2.02; 3.48]	1.91 [1.26; 2.56]
**Month 4**	3.56 [2.78; 4.33]	3.09 [2.36; 3.81]
**Month 6**	4.14 [3.38; 4.90]	4.12 [3.43; 4.80]
**Month 8**	4.64 [3.88; 5.40]	4.91 [4.37; 5.46]
**Month 10**	5.11 [4.37; 5.86]	5.50 [5.07; 5.93]
**Month 12**	5.69 [4.96; 6.43]	6.12 [5.68; 6.55]

The time courses of sensation of PHF were similar to those obtained for the intensity of pain ( Fig. 3 ; Table 3 ).

**Table:** **Table 3** Sensation of pressure, heaviness, or fullness – Difference from baseline at different time points (follow-up ITT; means and 95% CI).

Time Point	Former Menthacarin group (N=36)	Former placebo group (N=34)
**Day 29**	3.00 [2.27; 3.73]	2.06 [1.31; 2.81]
**Month 4**	3.42 [2.64; 4.19]	3.21 [2.34; 4.07]
**Month 6**	4.17 [3.49; 4.84]	3.94 [3.17; 4.71]
**Month 8**	4.56 [3.84; 5.27]	4.94 [4.35; 5.54]
**Month 10**	4.94 [4.27; 5.62]	5.56 [5.09; 6.02]
**Month 12**	5.56 [4.91; 6.20]	6.00 [5.50; 6.50]

**Fig. 5:**
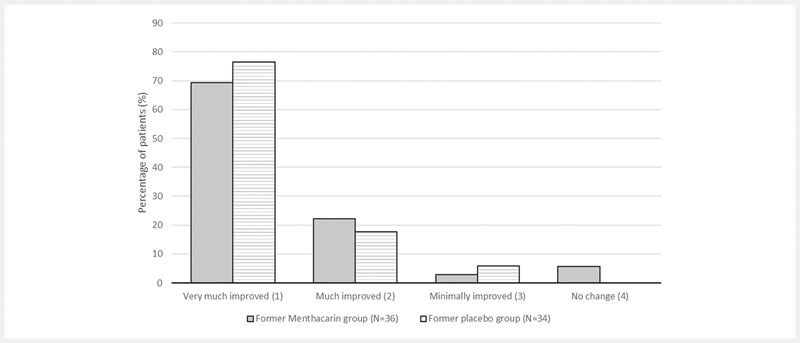
CGI item II – Global improvement at the end of follow-up, compared to the patient’s condition at baseline (follow-up ITT; percentage of patients). Note: Provided are the proportions of patients within the respective assessment categories observed at month 12 based on 36 patients in the former Menthacarin group and 34 patients in the former placebo group (treated with placebo until day 29, then switched to Menthacarin). Any patients with worsening were not reported.

With regard to the patients’ global improvement (CGI item II), 91.7% of the former Menthacarin patients (33/36) and 94.1% of the former placebo patients (32/34) were described as “much or very much improved” ( Fig. 5 ); at month 12, mean changes from baseline in the continuous analysis were 1.44 ± 0.81 points and 1.29 ± 0.58 points, respectively. Generally, the descriptive p-values derived from the of the 2-sided Wilcoxon-Mann-Whitney test did not show any significant differences between the 2 former treatment groups for any of the 3 primary outcome variables from month 4 onwards.

**Fig. 6:**
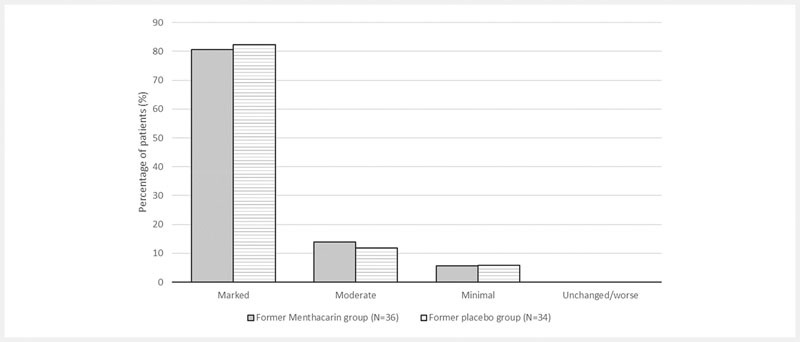
CGI item III – Therapeutic effect at the end of the follow-up (follow-up ITT; percentage of patients). Note: Provided are the proportions of patients within the respective assessment categories for the therapeutic effect observed at month 12 based on 36 patients in the former Menthacarin group and 34 patients in the former placebo group (treated with placebo until day 29, then switched to Menthacarin).

The results seen in the analyses of the main secondary outcome variables fully supported the findings from the 3 primary outcome variables. At month 12, the mean frequency of pain had decreased by 5.69 ± 2.08 points in the former Menthacarin group and by 5.91 ± 1.82 points in the former placebo group. The DDS showed a mean decrease of 27.6 ± 10.0 points in the former Menthacarin patients and of 27.1 ± 7.1 points in former placebo patients. Regarding the patients’ severity of illness (CGI item I), the mean value in the continuous analysis decreased from 4.08 ± 0.60 points at baseline to 1.69 ± 0.75 points at month 12 in the former Menthacarin group and from 4.15 ± 0.50 points to 1.71 ± 0.72 points in the former placebo group. The therapeutic effect (CGI item III) at month 12 ( Fig. 6 ) was evaluated as “marked” (very good) in 80.6% of former Menthacarin patients (day 29: 25.0%) and 82.4% of former placebo patients (day 29: 14.7%).

### Safety and tolerability

During the initial double-blind treatment period, a total of 4 patients of the follow-up population (3 patients in the Menthacarin group and 1 patient in the placebo group) experienced non-serious AEs that were either unrelated to the study or at least attributable to an aggravation of the disease under study. These events were (1patient each) “hemorrhoids” (moderate, resolved), “bronchitis” (mild, resolved), and “influenza-like symptoms” (moderate, resolved) in the Menthacarin group, and “pain in neck/shoulder” (mild, resolved) in the placebo group.

In the subsequent 11-month follow-up period, AEs were observed in 18 (48.6%) and 21 patients (60.0%) of the former Menthacarin group and placebo group, respectively (i.e., 39 patients, 54.2% with AEs overall).

Generally, the pattern of reported AEs covered a broad range of clinical conditions. “Musculo-skeletal system disorders” and “respiratory system disorders” were the most commonly involved SOCs among the 72 patients who entered the follow-up period (17 study patients, 23.6% each). At the preferred term level, AEs occurring in at least 5.0% of study patients were “bronchitis” (15.3%), “cervical pain” (13.9%), “tonsillitis” (5.6%), and “inflicted injury” (5.6%).

None of the documented AEs was serious, none was regarded as drug-related or had required premature treatment discontinuation, and apart from 1 patient with severe “vein disorder”, all events had a maximum intensity of mild or moderate at most. Laboratory analyses and vital sign monitoring did not point to relevant changes during the follow-up period.

## Discussion

The data from the double-blind, placebo-controlled phase of this trial over 29 days demonstrated for all 3 primary efficacy variables already at the first interim analysis that Menthacarin was superior to placebo in terms of FD symptom relief 15 with beneficial effects in both *H. pylori* -positive and *H. pylori* -negative patients 28 .

The extent of the observed pain relief during treatment with Menthacarin after the double-blind treatment period of the study was consistent with the results seen in other RCTs, in which Menthacarin was investigated for the treatment of FD 14 15 16 17 . Despite a barely predictable placebo response among patients with gastrointestinal disorders, which is reported to range between 20% and 60% 10 , all 3 placebo-controlled trials showed a statistically significant difference compared to placebo after 4 weeks of treatment. Moreover, the favorable treatments results seen in the NDI analyses separated by symptom subscores resembling the EPS and PDS umbrella terms according to Rome III suggested superior clinical efficacy of Menthacarin compared to placebo independently of the used classification system 17 . In addition, the concordance between the results based on the validated NDI and the VAS results seen in the study of Rich and colleagues 17 supported the validity of the earlier studies, in which the VAS was used as the primary assessment instrument.

Study data on the administration of Menthacarin for the treatment of FD in the long term are sparse. Among the 114 patients treated over 1 month in the RCT reported by Rich and colleagues 17 , 54 decided to continue their active (n=34) or placebo treatment (n=20) for an additional 8 weeks. The analyses at week 12 still showed statistically significant differences in favor of Menthacarin in the NDI subscores for pain and discomfort, respectively, as well as an improved quality of life compared with placebo 29 . In view of the lacking experience with Menthacarin in the long-term administration, an important finding of the current study was that the clinical improvements observed after 1 month of treatment in the former Menthacarin group were not only sustained, but improvements even progressed through month 12, while the former placebo patients had achieved similar improvements from month 6 onwards at the latest. Although the natural course of the disease is described as periodical with phases of slight or no symptoms alternating with periods of intensive complaints 9 , the continuously ongoing improvements in both patient groups through month 12 strongly suggested that the treatment with Menthacarin is suitable even for long-term administration.

In addition to the sustained treatment effects of Menthacarin, the current study demonstrated a positive risk/benefit ratio of Menthacarin, since also the long-term safety profile of Menthacarin was favorable. Safety observations during the treatment period of 1 month were consistent with those reported from the other RCTs 13 14 16 17 . Moreover, no serious AEs, obvious adverse drug reactions, or treatment-limiting AEs were reported through the 11 months of follow-up reported here. Notably, none of the reported AEs was related to gastrointestinal complaints, suggesting that the pre-existing FD was sufficiently controlled by continued treatment with Menthacarin. The AEs observed rather reflected the high incidence of musculoskeletal complaints and infections of the upper respiratory tract in a general, middle-aged outpatient population. The safety data obtained in this follow-up study thus indicate that continuous treatment of FD patients with Menthacarin over a period of up to 1 year is safe and well tolerated. Moreover, the observed medication compliance, a parameter which is generally considered to be important in long-term treatments 30 , lay within the range commonly defined in the literature as good (80 to 120% of the medication prescribed 31 . This shows that the treatment regimen (gastro-resistant capsule with twice-daily administration) was well accepted by the patients.

These findings were in line with the results of a systematic safety data review, in which study data of 3,144 patients and 58 healthy volunteers were included 32 . There were no serious AEs, and – apart from a slightly higher relative risk of eructation and skin sensitivity reactions – the AE patterns were similar in actively treated and placebo patients. A very good safety and tolerability of Menthacarin treatment was also reported from a 12-weeks placebo-controlled clinical trial in patients with irritable bowel syndrome 33 .

## Overall conclusions

The results of the current study corroborated the clinical importance of herbal drugs in general and of Menthacarin in particular for the management of FD with symptoms such as pain, cramps, bloating, and fullness, as it demonstrated a positive risk/benefit ratio of Menthacarin and suggests that – besides the symptomatic short-term treatment of EPS and PDS symptoms – Menthacarin is also helpful for long-term administration in FD patients, in whom a longer treatment duration is deemed required. This feature of Menthacarin appears to be of particular clinical relevance in view of the potentially severe side effects of alternative treatment options such as PPIs and prokinetics. Thus, due to proven efficacy and favorable safety, Menthacarin can be considered a drug of first choice in the short- or the long-term treatment of patients with FD of either subtype.

## Contributorsʼ Statement

MS made substantial contributions to the interpretation of data, revised the work critically for important intellectual content; and approved the version to be published. BS made substantial contributions to the interpretation of data, revised the work critically for important intellectual content and approved the version to be published. Both authors agree to be accountable for all aspects of the work in ensuring that questions related to the accuracy or integrity of any part of the work are appropriately investigated and resolved.
